# Untested assumptions: psychological research and credibility assessment in legal decision-making

**DOI:** 10.3402/ejpt.v6.27380

**Published:** 2015-05-19

**Authors:** Jane Herlihy, Stuart Turner

**Affiliations:** Centre for the Study of Emotion and Law, London, UK

**Keywords:** PTSD, refugee, asylum, sexual violence, decision-making

## Abstract

**Background:**

Trauma survivors often have to negotiate legal systems such as refugee status determination or the criminal justice system.

**Methods & results:**

We outline and discuss the contribution which research on trauma and related psychological processes can make to two particular areas of law where complex and difficult legal decisions must be made: in claims for refugee and humanitarian protection, and in reporting and prosecuting sexual assault in the criminal justice system.

**Conclusion:**

There is a breadth of psychological knowledge that, if correctly applied, would limit the inappropriate reliance on assumptions and myth in legal decision-making in these settings. Specific recommendations are made for further study.

Survivors of major trauma are more likely than most to find themselves having to deal with the law. This might arise, for example, in a criminal prosecution of a perpetrator; a claim for compensation; or an application for refuge, child protection, or an official investigation into a disaster. In all of these settings, there is a judicial spotlight on the validity of the survivor's account of what happened, in particular on the degree to which their emotional state has affected their ability to recall these events accurately.

There are often very different approaches taken by those coming at this problem from legal and psychological backgrounds. Maroney, a US academic lawyer, for example, suggests that the law is historically and structurally reliant on an assumption that rationality, not emotion, is what must be applied, and that emotion can only “muddy the water,” if not cleared away (Maroney, 2011). Although mental health professionals recognise that these emotional aspects cannot be “cleared away,” there remain striking difficulties in demonstrating how to work with these complexities in the furtherance of justice.

This paper will examine and discuss the contribution which research on trauma and related psychological processes can make to two particular areas of law—refugee law and the reporting and prosecution of sexual assault.

These areas of law have two key issues in common: the salience of the trauma experience, and the frequent absence of any objective evidence. For example, in cases of rape—where the issue concerns consent—almost always it is a case of one person's story against another's. Similarly in claims for asylum, someone with a history of detention and torture is unlikely to have any documentation to confirm their account. This means that decisions in both types of case rely heavily on an initial assessment of the credibility of the trauma history (Archambault & Lindsay, 2001; Herlihy & Turner, 2013; Jordan, 2004; Kelly, 2010; Noll, 2005). Key studies have shown how the two issues (trauma and credibility) can interact with undesirable results—setting those with posttraumatic stress disorder (PTSD) and related symptoms at a significant disadvantage. For example, in an analogue study of state decision-making about asylum seekers, Rogers, Fox, and Herlihy (2015) showed how the overlap between the ways in which we think people act when they are lying and the presentation of PTSD can lead to incorrect credibility judgements about some people with PTSD. Similarly, Hardy, Young, and Holmes (2009) demonstrated that women who reported higher peri-traumatic dissociation were more likely to report fragmentation of their memories of rape, and more likely to consider withdrawing their allegations for fear of being disbelieved.

This paper will first examine credibility assessment in decisions about people asking to be recognised as refugees (asylum seekers), highlighting the role that psychological science has to play, looking at decisions about adults, and then briefly noting the particular issues raised by young people seeking asylum.

We will then turn to the area of reporting and prosecuting sexual assault, based on our learning from our work with refugee law, drawing parallels and making recommendations for further research.

## Refugee law

In Europe, when making claims for state protection, or asylum, individuals have to convince a state and/or judicial decision maker that they are a person with “a well-founded fear of being persecuted for reasons of race, religion, nationality, membership of a particular social group, or political opinion” (United Nations, 1951). This is a most unusual legal definition carrying at its heart the emotional concept of well-founded fear. Whilst this definition is the basis of international treaty, states are allowed to construct their own procedures for recognising refugees. To guide them, the United Nations High Commissioner for Refugees (UNHCR) produces a (non-binding) handbook. This states that, “The relevant facts of the individual case will have to be furnished in the first place by the applicant himself.” It will then be up to the person charged with determining his or her status (the examiner) to assess the validity of any evidence and the credibility of the applicant's statements (UNHCR, 2011).

For many refugees, there is no evidence apart from the account they give and some background knowledge of their country of origin. States that carry out torture or other human rights abuses generally do not publish the fact. Asylum seekers rarely arrive with bundles of documents providing objective evidence of their claims. Typically, the decision has to be based on a combination of an analysis of the facts presented (do they meet this criterion) and, if they do, on the credibility of the applicant (can they be believed).

## A study of judicial assumptions in asylum decisions

This reliance on the assessment of credibility makes refugee status determination a particularly interesting area of law for psychological study. Given its life and death significance, it is also extremely important. A recent report on the asylum process in the United Kingdom quoted immigration judges as saying that their task was to rely on “common sense and experience” (Independent Asylum Commission, 2008) to decide the credibility of the people before them, the plausibility of the histories they provide, and the reliability of their testimonies.

In the United Kingdom, immigration judges have to produce a written determination setting out the basis of the claim, the law on which they rely, and then the decisions they reach, that together make up the basis of their final judgement. Herlihy, Gleeson, and Turner (2010) carried out a qualitative investigation of the assumptions made by UK judges in reaching their conclusions. Three major themes emerged. First, judges made assumptions about how (they believe) a credible claimant “would have behaved” in situations of fear or traumatic experience. For example, the account of the husband who “sent [his wife] to this country ahead of anyone in his own family, including his sister, who had been raped” was found to lack credibility. Second, those applying for asylum were judged on their behaviour in following the rules for the application process or in how they presented their evidence in court. For example, of a man alleging persecution on the grounds of his sexuality, from a country where homosexuality is illegal, the judge wrote, “the appellant denies having slept with the sponsor, which the sponsor [a UK citizen] says has occurred” (Herlihy et al., 2010, p. 360). These are questions about the cross-cultural communication in court and how well the “rules of conversation” (Grice, 1975) of the different cultures (of court and appellant) are understood by all individuals involved in the case. Third, assumptions about how the judges determined what was a truthful account emerged. This is where consistency in details reported on repeated questioning, early disclosure of all material facts (for example the assumption that rape survivors are able to disclose this experience to an official at first interview), and other lay assumptions about the nature of memory and disclosure were used to reach conclusions about the case.

What this study did was to open up these assumptions to definition and therefore to empirical investigation. Indeed in relation to some assumptions, for example, those concerning the nature of memory, there is already an extensive knowledge base (see Herlihy, Jobson, & Turner, 2012 for a review). Lay assumptions in the face of such scientific evidence may be completely unnecessary—but only if the understanding of memory is presented in a form accessible to these decision-makers. In other cases, clarifying assumptions in decision-making about adults seeking asylum has paved the way for their specific investigation—by psychologists (e.g., investigating memory for normal and traumatic events in adults), anthropologists, and sociolinguists.

## Assumptions in the asylum process about memory in adults

Seeking contradictions in an account of persecution given on different occasions was the most common “rule of thumb” identified in a study by Granhag et al. of members of the Swedish Migration Board (2005). However, for this to be a helpful discriminator of truthfulness and fabrication, it would also be essential that the survivor of trauma is always able to provide a clear and consistent narrative. This is one assumption that has been tested empirically (Herlihy, Scragg, & Turner, 2002) in programme refugees (who have not had to go through the asylum application process and for whom there was no motivation to deceive). Not only does this research show that, in line with the general body of research on recall, peripheral details of a traumatic account are more volatile than the central details, it also demonstrates that those with higher scores on a measure of PTSD symptoms and a longer delay between their interviews had more discrepancies in their accounts, suggesting that those who were most traumatised may have been most likely to be disbelieved on this criterion.

Similarly an assumption that a truthful account will also be detailed has been tested in a study showing higher levels of overgeneral memory in people seeking asylum with PTSD and depression (Graham, Herlihy, & Brewin, 2014). Overgeneral memory is particularly associated with depression, which is often poorly addressed in the asylum system (Wilson-Shaw, Pistrang, & Herlihy, 2012).

The assumption that timely and full disclosure of traumatic experiences is unproblematic in the asylum context has also been investigated (Bogner, Brewin, & Herlihy, 2009; Bogner, Herlihy, & Brewin, 2007), showing that, in line with a previous study of PTSD in refugees (Van Velsen, Gorst-Unsworth, & Turner, 1996), difficulty in disclosing material in an asylum interview is associated with PTSD avoidance symptoms, shame, and dissociation, and that these are higher in those with a history of sexual violence.

This emerging body of work therefore directly challenges some of the common assumptions being made about memory by decision-makers in the asylum process. Although fabricated accounts may be inconsistent, there are also often discrepancies in genuine trauma narratives. Some of the mechanisms involved in this finding have now been investigated, and the memory literature has been reviewed with reference to the asylum procedure (Herlihy et al., 2012), informing a United Nations report (UNHCR, 2013) on credibility assessment in European states, which will form the basis of future guidelines.

Of course, we accept that some people do set out to deceive decision-makers in a range of settings. It would be naïve to believe otherwise. Sometimes they may be aided by advice from legal representatives, others who have been through this process, or from the Internet. There is a sizeable literature on detecting deception, mostly aimed at improving police procedures, which has broadly concluded that we are all poor at detecting deception, but professional decision-makers are more confident in their abilities (Vrij, 2004), leading to the recommendation that training should aim to help decision-makers think more critically about their decision-making, increase awareness of the dangers of intuitive influences and “fast thinking” (Kahneman, 2011), and encourage the use of empirical cues (Porter & Ten Brinke, 2009). What we have demonstrated so far in this paper is simply that over-reliance by judges and other decision-makers on factors such as consistency in assessing credibility is similarly naïve and misplaced. The next big challenge is to try and see if it is possible to demonstrate any different patterns of discrepancy in those reporting truthful accounts from those deliberately intending to deceive—for example, does the degree of discrepancy matter; is frankly contradictory change in account more significant than reminiscence (recall of further detail on reflection); or is variability in situationally accessible memory, or sensory-bound representations (S-Reps) really less important than change in verbally accessible memory, or contextualised representations (C-Reps) (Brewin, Gregory, Lipton, & Burgess, 2010)?

## Assumptions in the asylum process about young people

The studies described so far all focus on adults seeking asylum. More than 12,000 unaccompanied children seek asylum throughout Europe every year,^1^ of which the majority are between 13 and 18 years old (Derluyn & Broekaert, 2008). These adolescents also have to show that they have a well-founded fear of persecution, in much the same way as adults. They face additional difficulties—to do with family separation, age assessment, and guardianship whilst they make their claim. An unpublished review by Given-Wilson of the psychological literature pertaining to young people seeking asylum has formed the basis of the UNHCR report The Heart of the Matter (UNHCR, 2014—see in particular Chapter 3). There is reason to believe that the same reliance on unfounded assumptions may apply to decision-making about children. For example, research has shown that adults may overestimate the mental capabilities of a child, particularly those who are more socially distant, for example, from a different culture (Bond, Omar, Mahmoud, & Bonser, 1990). Similarly psychological research examining other areas of law (e.g., concerning child sexual abuse) indicates that adult decision-makers are prone to making erroneous assumptions about a child's memory accuracy or capacity, likelihood to be truthful, and their ability to understand or respond in certain ways (Bruck, Ceci, & Hembrooke, 1998; Vrij, Akehurst, & Kinght, 2006). There is now an urgent need to investigate these matters specifically in relation to the application of refugee law.

## Credibility assessment in the prosecution of sexual assault

When a person is sexually assaulted, usually only the people concerned are present. Although there may be forensic evidence of sexual contact, in adults this is often insufficient, as it usually does not address the matter of consent. For this reason, the trauma narrative once again takes on a central role and the credibility of the survivor becomes crucial in the progress of any prosecution. Temkin and Krahé (2008) have argued that in all of the decision-making, from initial reporting to the police, through to courtroom conviction or acquittal, and sentencing, those making decisions are unduly influenced by their attitudes to rape and sexual assault. They present data from studies of law students, barristers, and community participants showing that decisions made by people who score highly on measures of adherence to rape myths are more likely to make judgements in line with those myths, rather than being driven by the evidence (Temkin & Krahé, 2008; see study 3). There has been much work done to articulate, publicise, and (try to) dispel the “myths of rape”^2^ but more is still needed.

Comparing the legal processes of reporting and prosecuting rape, against the descriptive and scientific psychological literature, a number of issues immediately become apparent. First internal consistency across different statements persists as a standard by which credibility is judged (Kelly, 2010). This not only assumes that recalled details of traumatic experiences will remain consistent, but it also assumes that the earliest account is the “true” account. This gives rise to the problematic situation whereby rape survivors who are willing to give evidence in a criminal prosecution are advised not to undergo any form of psychological therapy, in case questions by the therapist might lead the individual to develop a different version of what happened.^3^ Of course, this also assumes that if the person does not have therapy, there will be no change to the narrative. A recent pilot study included a woman who changed the date and day of a sexual assault. The researcher discussed the inconsistencies with the participant, who explained that the police record had stated “in the early hours of Tuesday morning,” which she had repeated at the early research interview, close in time to the police procedures, whereas, left to her own reflections, she now thought of it as something that happened as she left her house on a Monday night (Johnson, 2013). Thus (as in asylum seeker studies), reminiscence alone may lead to a change in the trauma narrative. A replication with rape survivors of our study of discrepancies in the accounts of asylum seekers is therefore relevant to one of the assumptions that is common in both areas: that memory for traumatic experiences is coherent and unchanging. Of course, it is not just memory that affects consistency of account in relation to sexual assault; other factors such as shame, fear, and posttraumatic avoidance do so as well. These factors substantially affect both sexes. However, we are also aware that men disclosing sexual assault are known to comprise another notoriously under-researched area (Coxell & King, 2010). Applying our understanding of the perceived (Sable, Danis, Mauzy, & Gallagher 2006) and reported (Easton, Saltzman, & Willis, 2013) barriers to disclosure for men within the asylum process is another area that needs to be pursued.

## Conclusion

This short paper sets out some of the current challenges at the interface between emotion and law. Although it may appear a daunting challenge to undertake research in this area, if the topic is broken down into a series of specific questions, it is in fact just like any other field of study. Indeed it is a sign of maturation in the field that such questions are being addressed (Turner, 2013). We founded the Centre for the Study of Emotion and Law^4^ as a focal point for the investigation and dissemination of these matters. We believe that justice is best served by finding and publicising the hard evidence on which better decision-making can take place.

## Supplementary Material

Untested assumptions: psychological research and credibility assessment in legal decision-making

Untested assumptions: psychological research and credibility assessment in legal decision-making

Untested assumptions: psychological research and credibility assessment in legal decision-making

Untested assumptions: psychological research and credibility assessment in legal decision-making

Untested assumptions: psychological research and credibility assessment in legal decision-making

Untested assumptions: psychological research and credibility assessment in legal decision-making

## Footnotes

1
www.epp.eurostat.ec.europa.eu/portal/page/portal/population/publications/migration_asylum

## Footnotes

2
See for example the UK Crown Prosecution Service at www.cps.gov.uk/news/articles/prosecuting_violence_against_women_and_girls_-_improving_culture_confidence_and_convictions/

## Footnotes

3 This was key in a widely reported case in the United Kingdom where a woman who was advised not to have any therapeutic support during a trial subsequently killed herself: www.theguardian.com/uk/2013/feb/09/frances-andrade-courts-son

## Footnotes

4
www.csel.org.uk offers an online research hub providing a resource for researchers in this field and a place to announce new work. Contact the authors for more details.

## References

[CIT0001] Archambault J, Lindsay S, Reuland M, Brito C. S, Carroll L (2001). Responding to non-stranger sexual violence. Solving crime and disorder problems current issues, police strategies and organizational tactics.

[CIT0002] Bogner D, Brewin C, Herlihy J (2009). Refugees’ experiences of Home Office interviews: A qualitative study on the disclosure of sensitive personal information. Journal of Ethnic and Migration Studies.

[CIT0003] Bogner D, Herlihy J, Brewin C (2007). Impact of sexual violence on disclosure during Home Office interviews. British Journal of Psychiatry.

[CIT0004] Bond C, Omar A, Mahmoud A, Bonser R (1990). Lie detection across cultures. Journal of Nonverbal Behavior.

[CIT0005] Brewin C, Gregory J. D, Lipton M, Burgess N (2010). Intrusive images in psychological disorders: Characteristics, neural mechanisms and treatment implications. Psychological Review.

[CIT0006] Bruck M, Ceci S. J, Hembrooke H (1998). Reliability and credibility of young children's reports. From research to policy and practice. American Psychologist.

[CIT0007] Coxell A. W, King M. B (2010). Male victims of rape and sexual abuse. Sexual and Relationship Therapy.

[CIT0008] Derluyn I, Broekaert E (2008). Unaccompanied refugee children and adolescents: The glaring contrast between a legal and a psychological perspective. International Journal of Law and Psychiatry.

[CIT0009] Easton S. D, Saltzman L. Y, Willis D. G (2013). “Would you tell under circumstances like that?”: Barriers to disclosure of child sexual abuse for men. Psychology of Men & Masculinity.

[CIT0010] Graham B, Herlihy J, Brewin C (2014). Overgeneral memory in asylum seekers and refugees. Journal of Behavior Therapy and Experimental Psychiatry.

[CIT0040] Granhag P. A, Stromwall L. A, Hartwig M (2005). Granting asylum or not? Migration Board personnel's beliefs about deception. Journal of Ethics and Migration Studies.

[CIT0011] Grice P, Cole P, Morgan J (1975). Logic and conversation. Syntax and semantics, 3: Speech acts.

[CIT0012] Hardy A, Young K, Holmes E. A (2009). Does trauma memory play a role in the experience of reporting sexual assault during police interviews? An exploratory study. Memory.

[CIT0013] Herlihy J, Gleeson K, Turner S (2010). What assumptions about human behaviour underlie asylum judgments?. International Journal of Refugee Law.

[CIT0014] Herlihy J, Jobson L, Turner S (2012). Just tell us what happened to you: Autobiographical memory and seeking asylum. Applied Cognitive Psychology.

[CIT0015] Herlihy J, Scragg P, Turner S (2002). Discrepancies in autobiographical memories: Implications for the assessment of asylum seekers: Repeated interviews study. British Medical Journal.

[CIT0016] Herlihy J, Turner S (2013). What do we know so far about emotion and refugee law?. Northern Ireland Legal Quarterly.

[CIT0017] Independent Asylum Commission (2008). Fit for purpose yet? The Independent Asylum Commission's interim findings.

[CIT0018] Johnson R (2013). The nature of memory after trauma: Examining memory consistency and fragmentation after rape and sexual assault.

[CIT0019] Jordan J (2004). Beyond belief? Police, rape and women's credibility. Criminal Justice.

[CIT0020] Kahneman D (2011). Thinking, fast and slow.

[CIT0021] Kelly L (2010). The (in)credible words of women: False allegations in European rape research. Violence Against Women.

[CIT0023] Maroney T (2011). Emotional regulation and judicial behavior. California Law Review.

[CIT0024] Noll G (2005). Proof, evidentiary assessment and credibility in asylum procedures.

[CIT0025] Porter S, Ten Brinke L (2009). Dangerous decisions: A theoretical framework for understanding how judges assess credibility in the courtroom. Legal and Criminological Psychology.

[CIT0026] Rogers H, Fox S, Herlihy J (2015). The importance of looking credible: The impact of the behavioural sequelae of post-traumatic stress disorder on the credibility of asylum-seekers. Psychology, Crime & Law.

[CIT0027] Sable M. R, Danis F, Mauzy D. L, Gallagher S. K (2006). Barriers to reporting sexual assault for women and men: Perspectives of college students. Journal of American College Health.

[CIT0028] Temkin J, Krahé B (2008). Sexual assault and the justice gap: A question of attitude.

[CIT0029] Turner S (2013). Psychotraumatology in Europe: A personal history. European Journal of Psychotraumatology.

[CIT0030] UNHCR (United Nations High Commissioner for Refugees) (2011). *Handbook and guidelines on procedures and criteria for determining refugee status under the 1951 convention and the 1967 protocol relating to the status of refugees*.

[CIT0031] UNHCR (United Nations High Commissioner for Refugees) (2013). Beyond proof: Credibility assessment in EU asylum systems. http://www.refworld.org/docid/519b1fb54.html.

[CIT0032] UNHCR (United Nations High Commissioner for Refugees) (2014). The heart of the matter—Assessing credibility when children apply for asylum in the European Union. http://www.refworld.org/docid/55014f434.html.

[CIT0033] UN General Assembly (1951). Convention relating to the status of refugees.

[CIT0034] Van Velsen C, Gorst-Unsworth C, Turner S (1996). Survivors of torture and organized violence: Demography and diagnosis. Journal of Traumatic Stress.

[CIT0035] Vrij A (2004). Why professionals fail to catch liars and how they can improve. Legal and Criminological Psychology.

[CIT0036] Vrij A, Akehurst L, Kinght S (2006). Police officers’, social workers’, teachers’ and the general public's beliefs about deception in children, adolescents and adults. Legal and Criminological Psychology.

[CIT0037] Wilson-Shaw L, Pistrang N, Herlihy J (2012). Non-clinicians’ judgments about asylum seekers’ mental health: How do legal representatives of asylum seekers decide when to request medico-legal reports?. European Journal of Psychotraumatology.

