# Supplementary material for: Untested assumptions: psychological research and credibility assessment in legal decision-making
Source: Eur J Psychotraumatol. 2015 May 19;6:10.3402/ejpt.v6.27380. doi: 10.3402/ejpt.v6.27380 (PMC4439408; doi:10.3402/ejpt.v6.27380)
Supplement: Untested assumptions: psychological research and credibility assessment in legal decision-making [file EJPT-6-27380-s001.pdf]

Hypothèses non vérifiées : la recherche psychologique et l'évaluation de la crédibilité dans la prise de décision juridique

Jane Herlihy & Stuart Turner

Contexte : les survivants de traumatismes doivent souvent négocier avec les systèmes juridiques en ce qui concerne par exemple la détermination du statut de réfugié ou le système de justice pénale.
Méthodes et résultats: Nous décrivons et discutons de la contribution que la recherche sur les traumatismes et les processus psychologiques connexes peut apporter à deux domaines particuliers du droit où des décisions juridiques complexes et difficiles doivent être prises : dans les demandes de statut de réfugié et la protection humanitaire, et dans les rapports et les poursuites des agressions sexuelles dans le système de justice pénale.
Conclusion : Il est un bagage de connaissances psychologique qui, s'il est correctement appliqué, limiterait le recours inapproprié à des hypothèses et mythes dans la prise de décision juridique de ces paramètres. Des recommandations spécifiques sont faites pour une étude plus approfondie.

Mots-clés: ESPT, réfugiés, asile, violences sexuelles, prise de décision

Citation: European Journal of Psychotraumatology 2015, **6**: 27380 - http://dx.doi.org/10.3402/ejpt.v6.27380
