# Supplementary material for: Untested assumptions: psychological research and credibility assessment in legal decision-making
Source: Eur J Psychotraumatol. 2015 May 19;6:10.3402/ejpt.v6.27380. doi: 10.3402/ejpt.v6.27380 (PMC4439408; doi:10.3402/ejpt.v6.27380)
Supplement: Untested assumptions: psychological research and credibility assessment in legal decision-making [file EJPT-6-27380-s002.pdf]

Ungetestete Annahmen: psychologische Forschung und Glaubwürdigkeitsbeurteilung in Rechtsentscheidungen

Jane Herlihy & Stuart Turner

Hintergrund: Trauma-Überlebende haben häufig mit dem Rechtssystem zu verhandeln, z. B. für den Asylstatus oder mit der Kriminal-Justiz.
Methoden und Ergebnisse: Wie unterstreichen und diskutieren den Betrag, den Forschung in Bezug auf Trauma und verbundene psychologische Prozesse macht und zwar hinsichtlich zweier spezifischer Rechtsfelder, in den komplexe und schwierige Rechtsentscheide zu treffen sind: in Asylfragen und Menschenrechtsangelegenheiten und im Berichten und Verfolgen von sexueller Gewalt in der Kriminaljustiz.
Schlußfolgerung: Es gibt eine Bandbreite psychologischen Wissens, welches im Falle korrekter Anwendung, die unsachgemäße Verschränkung von Annahmen und Mythen in der Entscheidungsfindung reduzieren würden. Spezifische Empfehlungen für weitere Studien werden gemacht.

Schlüsselwörter: PTBS, Flüchtling, Asyl, sexuelle Gewalt, Entscheidungsfindung

Citation: European Journal of Psychotraumatology 2015, **6**: 27380 - http://dx.doi.org/10.3402/ejpt.v6.27380
