# Supplementary material for: Untested assumptions: psychological research and credibility assessment in legal decision-making
Source: Eur J Psychotraumatol. 2015 May 19;6:10.3402/ejpt.v6.27380. doi: 10.3402/ejpt.v6.27380 (PMC4439408; doi:10.3402/ejpt.v6.27380)
Supplement: Untested assumptions: psychological research and credibility assessment in legal decision-making [file EJPT-6-27380-s003.pdf]

Teszteletlen feltevések: pszichológiai kutatás és a hihetőség kérdése jogi döntéshozatal során

Jane Herlihy & Stuart Turner

Háttér: Traumát túlélő személyek gyakran kerülnek jogi tárgyalások kereszttüzébe a menekültstátusz igénylése miatt, vagy büntetőeljárás keretein belül.
Módszer és eredmények: Két jogi procedúra kerül megvitatásra és az ezekkel kapcsolatos kutatási eredmények és pszichés folyamatok. Az egyik a menekültek védelemét igénylő folyamat, a másik a szexuális erőszak jelentése és az ezzel kapcsolatos vádemelés.
Következtetések: Széleskörű pszichológiai tudás áll rendelkezésünkre, hogy a téves feltevések és mítoszok elkerülhetők legyenek a döntéshozatali folyamatban. Konkrét javaslatok kerülnek bemutatásra.

Kulcsszavak: PTSD, menekült, menedékhely, szexuális erőszak, döntéshozatal

Citation: European Journal of Psychotraumatology 2015, **6**: 27380 - http://dx.doi.org/10.3402/ejpt.v6.27380
