# Supplementary material for: Untested assumptions: psychological research and credibility assessment in legal decision-making
Source: Eur J Psychotraumatol. 2015 May 19;6:10.3402/ejpt.v6.27380. doi: 10.3402/ejpt.v6.27380 (PMC4439408; doi:10.3402/ejpt.v6.27380)
Supplement: Untested assumptions: psychological research and credibility assessment in legal decision-making [file EJPT-6-27380-s004.pdf]

Supuestos no probados: investigación psicológica y evaluación de la credibilidad en la toma decisiones legales

Jane Herlihy & Stuart Turner

Antecedentes: Los sobrevivientes del trauma a menudo tienen que negociar en sistemas legales como la determinación del estado de refugiado o el sistema judicial criminal
Métodos y resultados: Delineamos y debatimos la contribución que puede hacer la investigación sobre trauma y los procesos psicológicos relacionados en dos campos concretos de la ley donde deben tomarse decisiones legales complejas y difíciles: en las demandas de protección humanitaria y de estado de refugiado y en denunciar y perseguir las agresiones sexuales en el sistema judicial criminal.
Conclusión: Existe una amplio conocimiento psicológico que, aplicado correctamente, limitaría la dependencia inapropiada de suposiciones y mitos en la toma de decisiones legales en estos entornos. Se hacen recomendaciones específicas para futuros estudios.

Palabras clave: TEPT, refugiado, asilo, violencia sexual, toma de decisiones

Citation: European Journal of Psychotraumatology 2015, **6**: 27380 - http://dx.doi.org/10.3402/ejpt.v6.27380
