# Supplementary material for: Untested assumptions: psychological research and credibility assessment in legal decision-making
Source: Eur J Psychotraumatol. 2015 May 19;6:10.3402/ejpt.v6.27380. doi: 10.3402/ejpt.v6.27380 (PMC4439408; doi:10.3402/ejpt.v6.27380)
Supplement: Untested assumptions: psychological research and credibility assessment in legal decision-making [file EJPT-6-27380-s005.pdf]

Test Edilmemiş Varsayımlar: psikolojik araştırma ve yasal karar vermede güvenilirlik

Jane Herlihy & Stuart Turner

Arkaplan: Travma mağdurları zaman zaman göçmen durumu belirlenmesi ya da kriminal adalet sistemi gibi yasal sistemlerle müzakere etmek zorunda kalırlar.
Metotlar & Sonuçlar: Karmaşık ve zor yasal kararların verilmesi gerektiği yasaların iki belirli alanına ilgili psikolojik süreçlerin ve travma üzerine yapılan araştırmaların katkısı tartışılmış ve taslağı çıkarılmıştır. Bu iki alan göçmen ve insani koruma için olan iddialarda ve kriminal yargı sisteminde cinsel saldırı kovuşturmasıdır.
Sonuç: Doğru uygulanırsa, bu gibi durumlarda yasal karar verme sürecindeki mitleri ve varsayımlara uygun olmayan inancı kısıtlayacak geniş bir psikolojik bilgi mevcuttur. Gelecek çalışmalar için belli öneriler verilmiştir.

Anahtar Kelimeler: TSSB, göçmen, iltica, cinsel saldırı, karar verme

Citation: European Journal of Psychotraumatology 2015, **6**: 27380 - http://dx.doi.org/10.3402/ejpt.v6.27380
