# Supplementary material for: Untested assumptions: psychological research and credibility assessment in legal decision-making
Source: Eur J Psychotraumatol. 2015 May 19;6:10.3402/ejpt.v6.27380. doi: 10.3402/ejpt.v6.27380 (PMC4439408; doi:10.3402/ejpt.v6.27380)
Supplement: Untested assumptions: psychological research and credibility assessment in legal decision-making [file EJPT-6-27380-s006.pdf]

Netestirane pretpostavke: psihološko istraživanje i procena kredibiliteta u donošenju odluka iz domena prava

Jane Herlihy & Stuart Turner

Uvod: Osobe koje su preživele traumu često moraju da se snalaze u pravnom sistemu, kao što je regulisanje izbegličkog statusa ili sistem krivičnog prava.
Metod & rezultati: Ističemo i naglašavamo udeo psiholoških istraživanja trauma i povezanih psiholoških procesa u dve oblasti prava u kojima se moraju donositi kompleksne i teške pravne odluke: u potraživanjima za izbegličku i humanitarnu zaštitu i u izveštavanju i krivičnom gonjenju seksualnih napada u sistemu krivičnog prava.
Zaključak: Postoji psihološko znanje koje, ako se pravilno primeni, može ograničiti neprimereno oslanjanje na pretpostavke i mitove u pravnom procenjivanju. Prikazane su specifične preporuke za dalja istraživanja.

Ključne reči: PTSP; izbeglice; azil; seksualno nasilje; donošenje odluka

Citation: European Journal of Psychotraumatology 2015, **6**: 27380 - http://dx.doi.org/10.3402/ejpt.v6.27380
